# Recovery of Proteins and Bioactive Peptides From Potato Peels

**DOI:** 10.1002/fsn3.71028

**Published:** 2025-10-03

**Authors:** Aytunga Arik Kibar, Özlem Aslan, Halil Dasgin, Emel Önder Fırat, Halil Rıza Avcı, Serhat Koçer, Fatih Tosun

**Affiliations:** ^1^ TÜBİTAK MAM, Climate and Life Sciences Food Technology Research Group Gebze Kocaeli Türkiye; ^2^ Gıda ve Yem Kontrol Merkez Araştırma Enstitüsü Müdürlüğü Osmangazi, Bursa Türkiye; ^3^ Tarla Bitkileri Merkez Araştırma Enstitüsü Müdürlüğü Bitkisel Gıdalar Araştırma Merkezi Yenimahalle Ankara Türkiye

**Keywords:** antidiabetic, antihypertensive, antioxidant, bioactive peptides, dietary fiber, plant protein, potato

## Abstract

Potato peels, a significant byproduct of the potato processing industry, hold immense potential for sustainable utilization due to their rich composition of proteins, bioactive peptides, dietary fibers, and phenolic compounds. These components not only present opportunities for functional food development but also align with the principles of a circular economy by reducing waste and creating value‐added products. This review provides a comprehensive synthesis of current knowledge on potato peel proteins and bioactive peptides, covering extraction and purification methods, health‐promoting properties, and technological applications. Recent advances in enzymatic hydrolysis and membrane separation are discussed, along with the functional properties and health benefits of derived peptides, including antioxidant, anti‐inflammatory, antihypertensive, and antidiabetic activities demonstrated in in vitro and in vivo models. While enzymatic production methods are well studied, alternative approaches such as autolysis and fermentation remain underexplored and merit further investigation. The review also addresses food safety concerns associated with glycoalkaloids and protease inhibitors present in potato peels. Despite current challenges—such as low protein content, bitter taste, and limited bioavailability—integrated valorization strategies can enhance their economic and functional potential. Overall, potato peel‐derived proteins and peptides emerge as promising candidates for the development of functional foods and nutraceuticals, with future research needed to unlock their full application potential.

## Graphical abstract

This review provides a comprehensive analysis of potato peel proteins and bioactive peptides, highlighting their extraction methods, health benefits, and potential applications in nutraceuticals and functional foods.

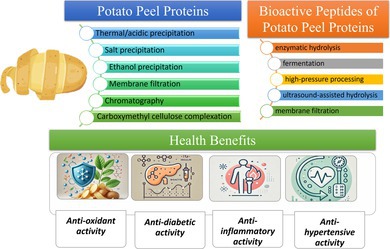

## 1 Introduction

The potato ( *Solanum tuberosum* ) ranks as the third most significant food crop globally and serves as a staple for billions of people. It plays a vital role in supporting the livelihoods of small‐scale farmers, offering a nutrient‐rich, low‐fat, and high‐fiber carbohydrate source abundant in antioxidants. Additionally, potatoes contribute less to greenhouse gas emissions compared to other major crops. Although its genetic roots trace back to South America, the potato is now cultivated on over 20 million hectares across 150 countries, producing a global yield of 359 million tonnes in 2020. By optimizing yields and utilizing traditional potato farming regions, global production could potentially increase to 500 million tonnes by 2025 and reach 750 million tonnes by 2030 (Dongyu 2022).

Approximately 50% of fresh potatoes are processed into four main potato products (chips, frozen, ready‐to‐eat, and starch) by industry (CIP 2024). The production of most potato products starts with a peeling step that can be applied via various methods such as abrasive peeling, steam, and high‐pressure water. Depending on the peeling method, the potato processing sector generates significant quantities of potato peel as a waste between 15% and 40% of the total weight of fresh potatoes (Hossain et al. 2015). This waste is typically exploited by the animal feed industry. However, it presents an appealing opportunity for upcycling into the food value chain due to it being considered as a valuable source of carbohydrates (Sampaio et al. 2020; Rodríguez‐Martínez et al. 2021). Potato peel contains a higher concentration of phenolic compounds compared to the potato flesh (Wu et al. 2012). Consequently, the extraction of bioactive compounds from potato peel holds promise for the development of innovative functional foods that promote health. Furthermore, its potential as a sustainable resource in integrated biorefineries could lead to diverse applications.

In recent years, plant‐based proteins have gained significant attention as an essential component in the development of sustainable food systems and nutrition strategies. The rising demand for alternative protein sources, driven by both environmental concerns and the global shift toward plant‐based diets, has led to increased research into underutilized agricultural byproducts. Among these options, potato peel, a byproduct of potato processing, stands out as a promising yet largely untapped source of high‐quality proteins and bioactive peptides.

Potato proteins, although present in lower quantities compared to carbohydrates, are of notable interest due to their exceptional nutritional quality. Recent studies have shown that potato peels contain a wide range of amino acids, with a total amino acid content of 42 ± 3 mg/g. Among these, the essential amino acids (EAA) are present at 15 ± 1 mg/g, including Val (3 ± 0.4 mg/g), Met (0.02 ± 0.00 mg/g), Ile (2.0 ± 0.1 mg/g), Leu (2.0 ± 0.2 mg/g), Phe (1.8 ± 0.04 mg/g), His (2.9 ± 0.08 mg/g), Thr (2.0 ± 0.2 mg/g), and Lys (2.0 ± 0.19 mg/g). Additionally, conditionally essential amino acids such as Arg (2.1 ± 0.04 mg/g), Cys (4.1 ± 0.01 mg/g), Gly (2.0 ± 0.3 mg/g), and Tyr (1.9 ± 0.01 mg/g) are also found (Zhang, Poojary, et al. 2021). This comprehensive amino acid profile, which includes all EAA, highlights the potential of potato peel proteins as a high‐quality plant‐based protein source suitable for human nutrition. Additionally, bioactive peptides derived from potato proteins have demonstrated potential health benefits, including antioxidant, anti‐inflammatory, and antihypertensive properties. These attributes position potato proteins as a valuable resource for developing functional foods aimed at improving human health.

As summarized in Table 1, the protein content of potato peels ranges between 8% and 17% on a dry weight basis, depending on the potato variety. In addition to protein, peels contain substantial amounts of carbohydrates and dietary fiber, which can also be fractionated and valorized individually through a biorefinery approach. This multifunctional valorization enhances the economic viability of utilizing potato peel in food and biotechnology sectors. Potato processing generates large quantities of peel as a waste byproduct, which, if effectively harnessed, could contribute to reducing waste while simultaneously providing a cost‐effective raw material for protein production. Incorporating proteins from potato peels into food products or utilizing them as raw materials in biorefineries supports the principles of a circular economy, fostering sustainability and efficient resource use.

**TABLE 1:** Macro and micro composition data of potato peel.

		References
**Macro components (dw)**		
*Chemical composition (%)*		
Moisture	85.06	Pathak et al. (2017)
Total carbohydrate	68.70	Pathak et al. (2017)
Total soluble sugar	1.00	Pathak et al. (2017)
Starch	52.14	Pathak et al. (2017)
Total dietary fiber	16.73	Joshi et al. (2020)
Cellulose	8.00	Jekayinfa et al. (2015)
Lignin	6.20	Jekayinfa et al. (2015)
Protein	8.00–17.1	Pathak et al. (2017) and Jekayinfa et al. (2015)
Lipids	2.60	Pathak et al. (2017)
Ash	6.34	Pathak et al. (2017)
**Micro components (dw)**		
*Minerals (mg/100 g)*		
Na	66.93	Joshi et al. (2020)
K	2764.39	Joshi et al. (2020)
Ca	200.80	Joshi et al. (2020)
Fe	21.69	Joshi et al. (2020)
Cu	2.48	Jekayinfa et al. (2015)
Mg	135	Vaitkevičienė (2019)
Zn	2.28	Jekayinfa et al. (2015)
P	1800	Jekayinfa et al. (2015)
*Phenolic content (mg GAE/g)*	4.3	Kähkönen et al. (1999)
*Vitamins (mg/100 g)*		
Thiamine	0.14	Joshi et al. (2020)
Riboflavin	0.25	Joshi et al. (2020)
Niacin	6.91	Joshi et al. (2020)
Ascorbic acid	76.31	Joshi et al. (2020)
*Glycoalkaloids (mg/100 g) (α‐solanine, α‐chaconine, and solanidine)*	1.26	Azizi et al. (2020)

Recently, there are a few relevant review articles published in 2023 and 2024 related to the extraction and potential of plant proteins and bioactive peptides; one example is a 2024 review that comprehensively explores bioactive peptides from natural sources, including their therapeutic potential and extraction methods like enzymatic hydrolysis, which could align well with the focus on bioactive peptides from potato peels. This review discusses the importance of bioactive peptides for human health and sustainability (Purohit et al. 2024). Another relevant review examines the bioactive compounds in potato peels, their extraction methods, and applications in the food industry (Jimenez‐Champi et al. 2023). This review highlights the nutritional benefits and bioactive properties of potato‐derived compounds, providing a foundation for their application in food systems and sustainable biorefineries. Additionally, a 2023 review discusses bioactive peptides, including their antioxidative, antimicrobial, and immunomodulatory properties (Najafian 2023). This article highlights the potential of bioactive peptides as key functional food ingredients, emphasizing their role in enhancing food quality and extending shelf life.

In conclusion, proteins and bioactive peptides derived from potato peels represent a highly promising yet underutilized resource for the development of functional food and nutraceutical applications. While a number of studies have addressed their extraction and biological properties, a focused and up‐to‐date synthesis of the scientific literature specifically on potato peel proteins and peptides is lacking. Moreover, the valorization of this agricultural byproduct is often discussed in broader contexts without in‐depth analysis of the protein fraction.

This review aims to fill this gap by critically evaluating the current knowledge on the extraction, purification, functional characterization, and health‐related properties of potato peel proteins and bioactive peptides. In doing so, it provides a focused perspective on their potential applications in sustainable food systems and highlights key challenges and opportunities for future research and industrial exploitation.

## 2 Composition of Potato Peel

For a thorough understanding of the physicochemical characteristics of potato peel, it's essential to examine both its physical and chemical composition. This understanding can aid in the development of eco‐friendly methods for utilizing potato peel (Javed et al. 2019). Table 1 provides an overview of the various macro‐ and micro‐components found in potato peel. Depending on where they are grown, the composition, types, color, and breed of potatoes (and consequently their peel) can differ (Javed et al. 2019).

Potato peel, primarily composed of starch, dietary fiber, and protein, possesses significant nutritional value. The most prevalent macronutrients in potato peel are carbohydrates, constituting 69–88 g/100 g dry weight (dw). Starch generally contributes to 30%–52% dw of its total carbohydrate content. Additionally, potato peels are rich in dietary fiber (~16.73%), which can offer various health benefits. These benefits include a cholesterol‐lowering effect and improved diabetic control (attributed to soluble dietary fiber), as well as regulation of intestinal health (due to insoluble dietary fiber) (Sampaio et al. 2020). The protein content of potato peels may vary, ranging from 1.2 to 4.4 g/100 g of fresh sample. Although the protein content of potato peels is relatively low compared to carbohydrates, their high biological value and functional potential—combined with the opportunity to valorize other fractions such as starch and fiber through an integrated biorefinery approach—make them a promising raw material for added‐value (Jimenez‐Champi et al. 2023). Choi et al. (2016) investigated the nutritional protein composition of potato peel and found a total crude protein concentration ranging from 9.52 to 10.58 g/100 g dry weight (dw). Additionally, they reported essential amino acid content ranging from 429 to 666 mg/100 g dw, a total free amino acid level ranging from 1383 to 2077 mg/100 g dw, and an asparagine range of 90.4–115.8 mg/g dw.

Potato peel also contains lipids, polyphenols, fatty acids, and phenolic acids, which play crucial roles in its antioxidant and antibacterial properties (Pathak et al. 2017). Phenolic compounds also exhibit other well‐established biological activities, encompassing apoptotic, anticarcinogenic, chemopreventive, and anti‐inflammatory properties (Rodríguez‐Martínez et al. 2021). The potato peel extract contains various phenolic acids, including gallic acid (58.6–63.0 mg/100 g), protocatechuic acid (216.0–256.0 mg/100 g), vanillic acid (43.0–48.0 mg/100 g), caffeic acid (278.0–296.0 mg/100 g), chlorogenic acid (753.0–821.3 mg/100 g), p‐hydroxybenzoic acid (82.0–87.0 mg/100 g), and p‐coumaric acid (41.8–45.6 mg/100 g) (Javed et al. 2019).

The mineral content in potato peel is typically between 0.9% and 1.6% in fresh weight (Jimenez‐Champi et al. 2023). In the case of vitamins, 100 g (on a fresh weight basis) of potato peels contains approximately 0.021 mg of thiamine, 0.038 mg of riboflavin, 1.033 mg of niacin, and 11.4 mg of ascorbic acid (Joshi et al. 2020).

In addition to the nutritional compounds mentioned above, potatoes also contain nitrogenous steroidal glycosides known as glycoalkaloids, which are recognized as stress metabolites. The primary glycoalkaloids found in potato peel are α‐solanine, α‐chaconine, and solanidine. The total concentration of α‐solanine and α‐chaconine in potato peel can vary significantly depending on factors such as the potato cultivar, light exposure, irradiation, storage conditions, and mechanical damage, ranging from 84 to 3526 ppm (Joshi et al. 2020).

## 3 Proteins and Bioactive Peptides of Potato Peels

### 3.1 Potato Peel Proteins

**FIGURE 1:**
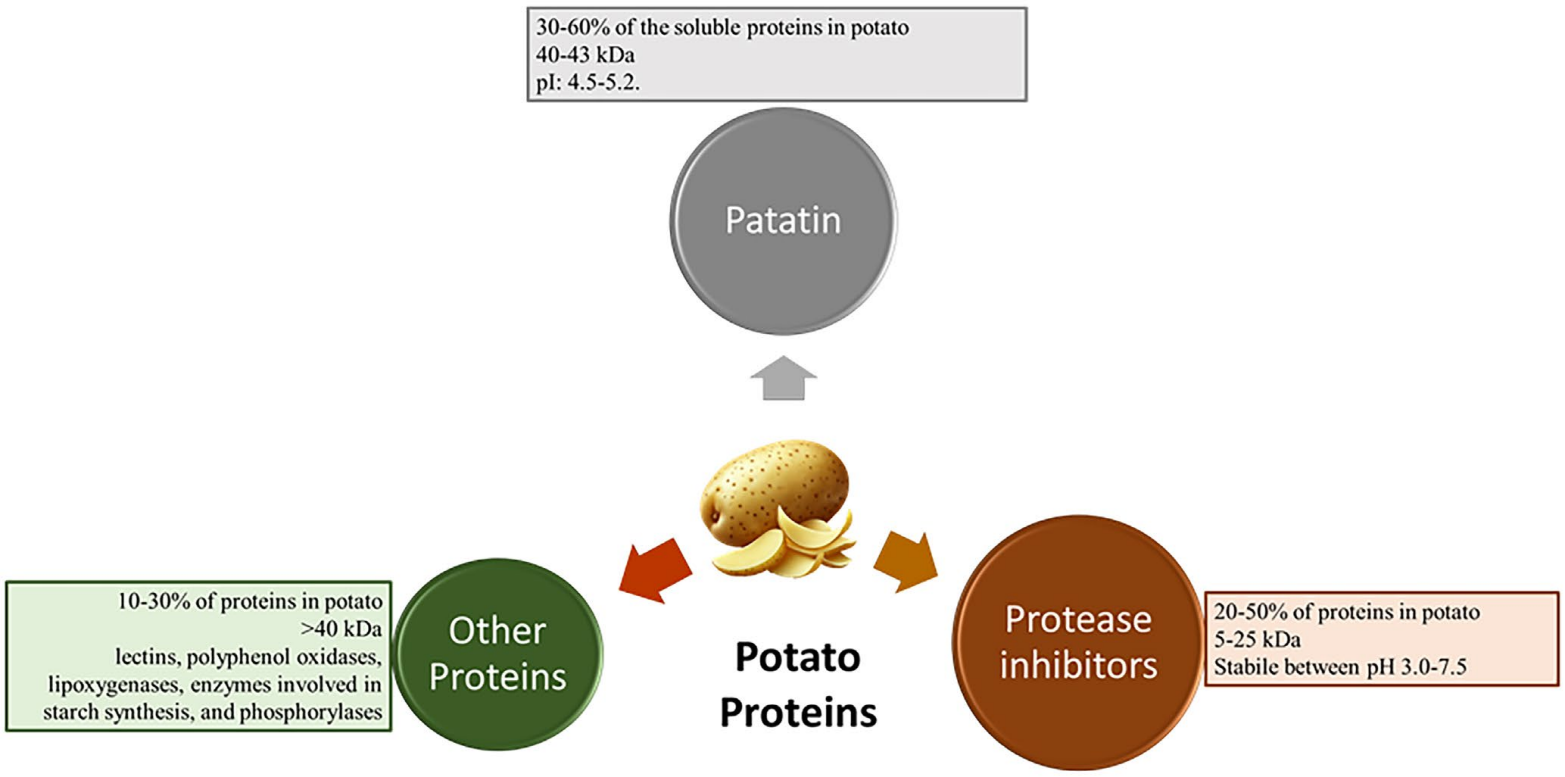
Proteins of potato.

Fresh potato tubers contain approximately 1.5%–4.0% protein. Since the pioneering work by Osborn and Campbell in 1896 on potato proteins, various separation and characterization studies have been conducted using techniques such as solubility fractionation, ion exchange (IE) chromatography, size exclusion chromatography, and electrophoresis (Shewry 2003). When potato proteins are separated by sodium dodecyl sulfate‐polyacrylamide gel electrophoresis (SDS‐PAGE) according to molecular size, several bands can be observed, while native polyacrylamide gel electrophoresis (Native‐PAGE) reveals numerous bands based on their charge differences (Hu et al. 2024; Huang et al. 2020). Potato proteins are divided into three main groups: patatins (30%–60%), protease inhibitors (20%–50%), and other proteins (10%–30%) (Figure 1) (Alting et al. 2011).

#### 3.1.1 Patatin

Patatin, the primary storage protein of potato tubers, was first isolated by Racusen and Foote using chromatographic methods and named after the potato itself. Due to its high accumulation in the tuber, patatin is considered a major storage protein. It forms a single protein band rich in hexosamine when fractionated by size exclusion chromatography but shows multiple bands when separated by SDS‐PAGE and isoelectric focusing electrophoresis (Alting et al. 2011).

Patatin is a glycoprotein, accounting for 30%–60% of the soluble proteins in potato tubers. Structurally, patatin consists of 366 amino acids and lacks distinct hydrophobic or hydrophilic clusters, with its positive and negative amino acids distributed irregularly throughout the sequence. It has a molecular weight of 40–43 kDa and an isoelectric point (pI) in the range of 4.5–5.2. Patatin contains about 5% neutral sugars and 1% hexosamine (Waglay and Karboune 2016b).

At neutral pH and room temperature, patatin exists as dimers with a molecular weight of approximately 80 kDa, held together by hydrophobic interactions. It also has a high degree of secondary structure, consisting of 35% α‐helix, β‐sheets, and 15% random coils (Bhutto et al. 2024).

Patatin has a low denaturation temperature (60°C at pH 7.0) and is highly sensitive to pH changes. It loses its three‐dimensional structure at pH 4.5 or lower. In addition to its structural characteristics, patatin exhibits broad non‐specific lipid acyl hydrolase, phospholipase A2, and lipid acyl transferase activities, which significantly affect its functional properties (Hussain et al. 2021). It has also been reported to show β‐1,2‐xylosidase activity and to participate in plant defense mechanisms against pathogenic fungi through β‐1,3‐glucanase activity (González‐Pérez and Arellano 2009).

#### 3.1.2 Protease Inhibitors

Protease inhibitors found in potato tubers serve as storage proteins rich in sulfur‐containing amino acids. These proteins regulate protease activity in the tuber and have various physiological roles in protecting the potato from pathogens. This group of proteins, with molecular weights ranging from 5 to 25 kDa, constitutes 20%–50% of the total potato protein content (Pouvreau et al. 2003).

They are more heterogeneous than patatin and are classified based on molecular weight, isoelectric point, and enzyme inhibition activity into seven subgroups: potato inhibitor‐I, potato inhibitor‐II, potato cysteine protease inhibitors, potato aspartic protease inhibitors, potato Kunitz protease inhibitors, other serine protease inhibitors, and potato carboxypeptidase inhibitors (Pouvreau et al. 2003).

Potato protease inhibitors exhibit a broad inhibition spectrum, with all but the carboxypeptidase inhibitors capable of inhibiting trypsin and/or chymotrypsin (Srikanth and Chen 2016). In addition to inhibiting digestive enzymes, protease inhibitors possess anticarcinogenic properties and can stimulate the production of cholecystokinin (Kennedy 1998). Due to their protective disulfide bonds, these proteins retain their three‐dimensional structures over a pH range of 3.0–7.5 (Srikanth and Chen 2016).

#### 3.1.3 Other Proteins

Proteins that are neither patatins nor protease inhibitors fall into this category. These proteins make up 10%–30% of the total potato protein content. They have molecular weights larger than 40 kDa and include lectins, polyphenol oxidases, lipoxygenases, enzymes involved in starch synthesis, and phosphorylases (Waglay and Karboune 2016b).

### 3.2 Extraction and Isolation of Potato Protein

Potato protein can be obtained from potato tubers or potato byproducts. When one metric ton of potatoes is processed into starch, it generates 5–12 cubic meters of potato juice. This juice contains between 300 and 410 g of protein per kilogram of total solids, representing 20%–60% of the proteins found in the potato tuber; it also comprises sugars (15.8%), organic acids (13.2%), minerals such as potassium and phosphorus (12.2%), other components (10.1%), lipids (2.2%), and minor phenolic acids like chlorogenic and caffeic acids (0.5%). The supernatant, obtained by centrifuging the potato juice, can be utilized for protein extraction (van Koningsveld et al. 2002; Løkra and Strætkvern 2009; Wojnowska et al. 1982). However, extracting protein from potato juice (PJ) is a challenging process due to its complex structure and high water content (Miedzianka et al. 2012). Techniques such as ultrafiltration, reverse osmosis, or fractional precipitation can be employed to increase the concentration of potato protein (Knorr et al. 1977). In industrial settings, the most common methods for protein recovery from PFJ typically involve heat coagulation and acid precipitation. However, the application of heat and acid denatures the proteins, causing them to lose their functional properties such as foaming, emulsification, or water and oil retention, thereby reducing their suitability for food applications (Boye et al. 2010; Miedzianka et al. 2012). Special methods are required to preserve the functional properties of proteins and prevent denaturation. As shown in Table 2, many methods have been investigated to isolate native proteins, such as salt precipitation, ammonium sulfate precipitation using organic solvents, complexation with carboxymethyl cellulose (CMC), chromatographic techniques, ultrafiltration, enzyme‐based isolation, and combinations of these methods (Zhang et al. 2017).

**TABLE 2:** Potato protein extraction and isolation methods.

The raw material/biomass	Method/precipitation agent	Yield	Advantages/drawbacks	References
Sweet potato peel	Salt precipitation/CaCl_2_	32.0% (w/w)	Low yield, low purification rates	Maloney et al. (2012)
PJ	Thermal/acidic precipitation	90.2% (w/w)	High yield/lost in functional properties, costly, low purification rates, sensory defects, potential hazards to the environment	Knorr et al. (1977) and Waglay et al. (2014)
	Acidic precipitation	64.7% (w/w)	Cost‐effective/moderate yield, lost in functional properties, low purification rates, sensory defects, potential hazards to the environment	Knorr et al. (1977) and Waglay et al. (2014)
	Salt precipitation/FeCl_3_	57.4%–75.2% (w/w)	High purification rates/moderate yield, lost in functional properties	Knorr et al. (1977) and Waglay et al. (2014)
	Salt precipitation/MnCl_2_	16.8% (w/w)	Enriches protease inhibitors/low yield, low purification rates	Waglay et al. (2014)
	Salt precipitation/(NH_4_)_2_SO_4_	70.1%–98.6% (w/w)	High yield, high purification rates, good functional properties/longer process, costly	van Koningsveld et al. (2001) and Waglay et al. (2014)
	Carboxymethyl cellulose complexation	23.6%–75.3% (w/w)	Higher purification rates/Moderate yield, lost in functional properties in low pH	Vikelouda and Kiosseoglou (2004) and Waglay et al. (2014)
	Ethanol precipitation	30.7%–55.2% (w/w)	Higher purification rates, simple to use, high nutritional value/moderate yield, costly	Bártová and Bárta (2009) and Waglay et al. (2014)
	Chromotography	37%–82% (w/w)	High separation efficiency and purity/challenges in scalability, costly	Andersson et al. (2008), Bhutto et al. (2024), Løkra et al. (2008) and Strætkvern et al. (1998)
	Membrane filtration	75%–80% (purity)	High yield and purity, gentle operating/sensory defects, presence of antinutritional factors, membrane fouling	Oosten (1976), Schmidt et al. (2016) and Zwijnenberg et al. (2002)
	Enzyme‐based isolation	63%–75% (w/w)	High yield, environment friendly/costly, challenges in scalability	Waglay et al. (2016)

Abbreviation: PJ, Potato Juice.

#### 3.2.1 Thermal/Acidic Precipitation

Acid and heat coagulation methods, whether used together or separately, have long been employed for protein recovery (Knorr et al. 1977). Historically, these methods have been utilized to extract proteins from PFJ, reducing pollution and costs. The pH was specifically adjusted to a range of 3.5–4.5 using hydrochloric acid. The separated juice was then heated with steam to a temperature above 90°C, followed by centrifugation to collect the precipitated potato protein. The potato protein concentrate was then obtained through spray drying. Heat coagulation is prevalent in the potato starch industry, but temperatures exceeding 90°C are commonly required, leading to economic drawbacks and rendering potato proteins insoluble, limiting their application in other industries (Knorr et al. 1977). Waglay et al. (2014) demonstrated that combining thermal and acidic techniques yields high protein quantities but low purification rates. Proteins recovered through acidic or heat coagulation from PFJ often exhibit a dark color and strong cooked flavor due to the harsh conditions, making them suitable for animal feed applications (Waglay et al. 2014).

#### 3.2.2 Salt Precipitation

One of the methods for isolating potato proteins involves the use of salts, which form complexes between protein and metal ions (Waglay et al. 2014). FeCl_3_ is the most commonly used salt for precipitating potato proteins from PFJ (Knorr 1982). Knorr et al. (1977) demonstrated that FeCl_3_ effectively coagulates potato proteins from PFJ, similar to acid and heat coagulation. Ferric chloride precipitation is a straightforward technique for protein precipitation, with higher FeCl_3_ concentrations resulting in increased protein recovery. However, a drawback of this method arises during protein content measurement post‐extraction, as the proteins precipitated with FeCl_3_ require a chelating agent to become soluble. Moreover, the high ferric ion content interferes with chelating agents, complicating protein determination using methods such as Bradford or BCA (Bártová and Bárta 2009). Although this method may not be directly suitable for food‐grade applications due to residual iron content, proper downstream processing can reduce salt levels to meet food safety standards. In other research on extracting proteins from sweet potato peels, a method was utilized where the peels were initially mixed with a saline solution to dissolve the proteins and then precipitated using CaCl_2_ and NaCl. Their findings revealed that the optimal yield was obtained when blanched peels were mixed with 59.7 mL of 0.025 mM NaCl per gram of peel and subsequently precipitated with 6.8 mM CaCl_2_. This study suggests that valuable proteins can potentially be extracted from sweet potato peels (Maloney et al. 2012).

Ammonium sulfate ([NH_4_]_2_SO_4_) precipitation is a commonly used technique for extracting proteins based on differences in solubility. This technique is also considered one of the most promising approaches for achieving high yields of non‐denatured proteins with exceptional purity. It also yields a significant amount of patatin compared to other methods (van Koningsveld et al. 2001). Bártová and Bárta (2009) observed an increase in the recovery yield of potato proteins with rising concentration during ammonium sulfate precipitation, whereas Waglay et al. (2014) noted the opposite trend, reaching a peak yield of 98% depending on concentration.

#### 3.2.3 Ethanol Precipitation

Ethanol precipitation is another method used to isolate potato proteins by disrupting protein–protein interactions (Bártová and Bárta 2009). In a study by Bártová and Bárta (2009), they compared the use of ethanol and ferric chloride as protein precipitators in industrial potato fruit juice in terms of yield, solubility, and the nutritional quality of the isolated protein concentrates. Both ethanol and ferric chloride significantly reduced the unwanted glycoalkaloid and potassium content in the protein concentrates. Ethanol precipitation was found to be more advantageous due to its ease of application and better preservation of the enzymatic activities of patatin proteins.

#### 3.2.4 Carboxymethyl Cellulose Complexation

Polysaccharide precipitating agents, like carboxymethyl cellulose (CMC), effectively precipitate proteins in lower pH conditions (Kong et al. 2015). When added in low CMC‐to‐protein ratios, CMC causes potato protein to coagulate, facilitating its easy recovery via centrifugation. Factors such as environmental pH, the ionic strength of proteins and polysaccharides, net charges of species, and molecular size, shape, and interactions influence precipitate formation (Vikelouda and Kiosseoglou 2004). An acidic environment may compromise the functional characteristics of proteins. Nonetheless, research conducted by Vikelouda and Kiosseoglou (2004) demonstrated that altering the pH to 2.5 impacts various functional characteristics of the proteins. According to this study, protein precipitates complexed with CMC were observed to have adequate solubility and foaming properties (Vikelouda and Kiosseoglou 2004). According to Partsia and Kiosseoglou (2001), potato protein isolated using CMC demonstrates satisfactory solubility.

#### 3.2.5 Chromatography

Chromatography is a widely utilized separation technique in the food industry, employed for the isolation and purification of food components such as proteins based on their sizes (Boxi et al. 2020). It is generally used after pretreatment steps that remove solids, fiber, or other interfering compounds to enhance separation efficiency and reduce column fouling (Løkra et al. 2008; Strætkvern et al. 1998). It demonstrates a notably high separation efficiency, particularly in the extraction of potato protein, notably for patatin and protease inhibitors. Expanded bed adsorption (EBA) and ion exchange (IEX) are commonly used separation techniques. Other methods include affinity chromatography, ion exchange (IE) chromatography, hydrophobic interaction chromatography (HIC), expanded bed adsorption (EBA) chromatography, and simulated moving bed (SMB) chromatography (Hussain et al. 2021). In research, ion exchange (IE) and hydrophobic interaction (HIC) chromatography have been utilized to recover potato proteins with desirable attributes such as light color, low glycoalkaloid content, and minimal polyphenol oxidase activity (Schmidt et al. 2017). A study indicates that 75% of the protein can be recovered by passing potato juice through ion exchange columns (Hussain et al. 2021). For the recovery of food‐grade protein preparations, expanded bed adsorption (EBA) has been identified as a suitable method for eliminating fiber, minerals, and pigments from potato fruit water (Løkra et al. 2008). Previous studies utilized the EBA method to isolate patatin with a mixed‐mode adsorbent at a pH of 7.5, achieving a 50% recovery rate (Strætkvern et al. 1998). When using the mixed standard mode resin (a hetero‐functional adsorbent ligand) at a pH of 6.0–6.5, the purity of the potato protein concentrate was approximately 80%. Additionally, the protein dried by atmospheric freeze‐drying demonstrated good functional qualities, such as emulsification and solubility (Løkra et al. 2008). Pilot‐scale studies on the recovery of potato proteins using chromatographic techniques have explored the utility of expanded bed adsorption for the recovery of patatin, achieving recovery rates ranging from 37% to 50% (Løkra et al. 2008). Inversely, simulated moving bed chromatography demonstrated an 82% protein recovery rate, albeit with decreasing extract purity with each pass through the process (Andersson et al. 2008).

Chromatography techniques used for extracting potato proteins ensure high separation efficiency and purity, allowing for the isolation of specific protein components. These methods offer exceptional resolution, making them ideal for applications that require well‐defined and pure protein fractions (Bhutto et al. 2024). Despite the promising results of chromatographic techniques, they have disadvantages such as the difficulty of implementing them on an industrial scale and the high investment costs involved. Industrial application is particularly challenging due to limitations such as the degradation of high‐density resins and the loss of binding sites over time, reducing the efficiency of column separation (Strætkvern et al. 1998).

#### 3.2.6 Membrane Filtration

Potential procedures without heat treatment include membrane filtrations such as reverse osmosis or ultrafiltration (van Koningsveld et al. 2002). Membrane filtration operates under pressure, where a semi‐permeable membrane retains large molecules (Saltik et al. 2016). Due to its gentle operating system, it is preferred for industrial applications. However, during reverse osmosis, proteins may develop a salty and bitter taste, as it tends to retain minerals and low molecular weight compounds, which may impart undesirable sensory characteristics, thereby reducing the functionality of the final product (Strætkvern and Schwarz 2011). Ultrafiltration followed by diafiltration shows more promise, as it can produce proteins of higher quality and yields of up to 50% (van Koningsveld et al. 2002). In a study, a plate ultrafiltration membrane was utilized to recover potato protein from PFJ at temperatures of 30°C–35°C, achieving a high purity of 75%–80% (Oosten 1976). In another study, ultrafiltration combined with additional diafiltration successfully yielded potato proteins with desirable emulsifying and foaming properties. Nevertheless, the final protein sample exhibited an undesirable brown color due to the presence of polyphenol complexes in the end product (Schmidt et al. 2016). Unfortunately, these techniques face limitations such as membrane fouling during large‐scale processing and the presence of antinutritional factors like protease inhibitors or glycoalkaloids. This may be partly due to the retention of low molecular weight compounds and some protein–phenolic or glycoalkaloid complexes during these processes (Strætkvern and Schwarz 2011; Zwijnenberg et al. 2002).

#### 3.2.7 Enzyme‐Based Isolation

A novel enzymatic technique has proven to be efficient in extracting functional proteins from potato pulp (Waglay et al. 2016). This method involves two main steps: initially, removing starch using the enzyme Termamyl, followed by breaking down the cell wall pectin network with glycosyl‐hydrolases to enhance protein retrieval (Waglay and Karboune 2017). A study discovered that starch removal was essential for the effectiveness of this enzymatic approach, leading to an increase in protein recovery from 47% to 63%–75% (Waglay et al. 2016). In another study, they scaled up a process to a pilot‐scale setting to isolate potato proteins from simulated byproducts of potato juice and pulp. The protein concentrate obtained post‐treatment with a multi‐enzymatic system showed statistically significant improvements in techno‐functionality, particularly in terms of the emulsifying activity index (Waglay et al. 2019). The enzymatic method can separate and break down PPs into components such as patatin and PIs with minimal negative effects. This technique is promising and environmentally friendly, providing practical solutions for industries aiming for efficient and sustainable protein extraction. Although there are challenges in optimizing the process for maximum yields, cost‐efficiency, and scalability, it takes advantage of renewable potato properties and reduces chemical use. Recommendations include refining techniques and enhancing enzyme recovery, which could potentially transform PP production and improve the sustainability of the protein supply chain (Bhutto et al. 2024).

### 3.3 Bioactive Peptides of Potato Proteins

Bioactive peptides are small fragments of food proteins that are primarily composed of 2–20 amino acid subunits, with a molecular weight of < 3 kDa. These peptides, which are latent within the parent protein structure, become active when released through enzymatic hydrolysis, fermentation, or gastrointestinal digestion (Chalamaiah et al. 2018). Recently, bioactive peptides derived from food proteins, including potato proteins, have gained attention due to their numerous properties and applications. They exhibit various biological activities influenced by their amino acid composition, sequence, and chemical structure (Lemes et al. 2016). Specifically, bioactive peptides obtained from plant byproducts, such as those derived from potatoes, not only contribute to health‐related functions but also enhance the technological properties of foods (Martínez‐Medina et al. 2021; Rizzello et al. 2016).

Potato proteins remain a relatively untapped resource for bioactive peptides, offering significant potential for creating innovative functional foods. The protein composition of potatoes, mainly consisting of globulins, albumins, and protease inhibitors, exhibits notable bioactive properties, making them an excellent candidate for further research and application in functional food development and nutraceutical products (Toldrá et al. 2018).

#### 3.3.1 Production and Extraction of Bioactive Peptides From Potato Proteins

Bioactive peptides can be produced from potato proteins using various methods. These methods are enzymatic synthesis, chemical synthesis, and autolysis (Kamnrdpetch et al. 2007; Mäkinen et al. 2016; Shwaiki et al. 2020). Enzymatic synthesis is performed using endoproteases, exoproteases, or a combination of both (Kamnrdpetch et al. 2007; Kudo et al. 2009; Waglay and Karboune 2016a). Examples of enzymes used for the enzymatic synthesis of potato‐derived bioactive peptides include Alcalase, Flavourzyme, Novo Pro‐D, Corolase, Pancreatin, Amano‐P, Thermoase PC10F, Protease S, Proleather FG‐F, Papain, and Pepsin (Ishiguro et al. 2012; Kamnrdpetch et al. 2007; Kudo et al. 2009; Udenigwe et al. 2016; Waglay and Karboune 2016a). It was shown that the combination of two enzymes enhanced the degree of hydrolysis (Kamnrdpetch et al. 2007).

In recent years, several techniques have also been used in combination with the enzymatic treatment. Some of these techniques include hydrostatic high‐pressure processing, microwave, thermal (e.g., boiling, steaming, autoclaving), ultrasound, and radio frequency treatments (Nazir et al. 2020; Zhang et al. 2019, 2020). Studies showed that the combination of these treatments with enzymatic hydrolysis increased the degree of hydrolysis and the production of low molecular weight peptides (Nazir et al. 2020; Zhang et al. 2019, 2020). These improvements are likely due to conformational changes, unfolding of protein structures, and protein denaturation caused by the applied treatment (Nazir et al. 2020; Zhang et al. 2019).

Another commonly employed method for obtaining potato‐derived peptide is chemical synthesis. Snakin‐1, FGER, FDRR, and FGERR are some examples of chemically synthesized potato‐derived peptides (Kudo et al. 2009; Shwaiki et al. 2020).

Besides enzymatic and chemical synthesis, autolysis treatment has also been performed to produce potato‐derived bioactive peptides. Although autolysis is cost‐effective, it has been used less frequently in comparison to enzymatic synthesis (Mäkinen et al. 2016). Another cost‐effective method for generating bioactive peptides is fermentation. However, to date, no studies have employed the fermentation process to obtain bioactive peptides from potato proteins.

#### 3.3.2 Advances in Purification and Characterization

After extraction, the purification and characterization of bioactive peptides are essential to guarantee their effectiveness and safety for use in food and nutraceutical products. Protein hydrolysis is conducted under controlled conditions, and the resulting hydrolysates are evaluated for their biological activity. These hydrolysates undergo sequential fractionation, often involving ultrafiltration with various molecular weight cut‐off membranes to concentrate bioactive fractions. Additionally, liquid chromatography techniques are commonly utilized to purify peptides, ensuring the desired bioactive properties are retained (Martínez‐Medina et al. 2021; Rizzello et al. 2016; Toldrá et al. 2018).

However, the purification process can be exhaustive and time‐consuming, especially given the complexity of the system. An alternative approach involves selecting the protein source and enzyme, followed by simultaneous evaluation of the resulting hydrolysate for various biological activities. This can be complemented with peptidomic analysis, where peptide sequences are compared against bioactive peptide databases. Additionally, in silico methods provide a valuable tool for simulating the hydrolysis of food proteins, enabling the prediction and assessment of the bioactivities of resulting peptides through bioinformatics techniques (Toldrá et al. 2018; Zhang, Tian, et al. 2021).

The assessment of in vitro bioactivities often serves as a foundation for identifying novel peptide sequences, making it a critical area of research with significant scientific contributions. However, the bioactive properties of these peptides should ideally be validated through studies involving human or animal models. The following sections focus on the impact of certain food protein‐derived peptides on in vivo responses, which are typically evaluated using high‐purity peptides, often synthesized chemically. A common challenge in this field lies in advancing the processing of protein hydrolysates to achieve high‐yield peptide products with improved bioactivity, a crucial step for their application in functional foods and nutraceuticals (Martínez‐Medina et al. 2021; Rizzello et al. 2016).

#### 3.3.3 Potential Health Benefits of Potato Protein‐Derived Bioactive Peptides

Biological activities of bioactive peptides vary according to the type, sequence, and molecular weight of amino acids. Health effects, evaluation, and treatment models and amino acid sequences of potato‐originated peptides are presented in Table 3.

**TABLE 3:** Health effects of potato protein, potato protein hydrolysate, and potato protein peptides.

Potato protein, peptide name or sequence	Protease used for protein hydrolysis/the method of obtaining peptides	Health effect	Assays related to health effects	Evaluation model‐outcome	References
Patatin	Chromatography on DEAE‐cellulose and Con A Sepharose	Antioxidant	DPPH, peroxynitrite‐mediated dihydrorhodamine 123 oxidation	In vitro tests—displayed scavenging activity against DPPH radicals and protected against peroxynitrite‐mediated dihydrorhodamine 123 oxidations	Liu et al. (2003)
Potato protein hydrolysate (Pepsin) Potato protein hydrolysate (Pepsin + Pancreatin)	Pepsin/Pepsin + Pancreatin	Antioxidant	Fe (III) reducing capacity, Fe (II) chelating capacity, in vitro glutathione oxidation assay	In vitro tests—displayed Fe (III) reducing capacity and Fe (II) chelating capacity Protected and regenerated GSH	Udenigwe et al. (2016)
Potato protein isolate hydrolysate	Bromelain, Neutrase, and Flavourzyme	Antioxidant	(ABTS) radical‐scavenging assay, cell‐viability assay	Cell culture model—Protected C2C12 murine myoblast cell line from H_2_O_2_ oxidation	Chang et al. (2020)
YFE	Alcalase	Antioxidant	Measurement of cellular MDA and protein carbonyl groups, antioxidant enzymes	Cell culture model—Protected Caco‐2 cell line from H_2_O_2_ oxidation	Wu et al. (2020)
FGER, FDRR, FGERR	Pancreatin and Amano‐P	Antioxidant	Ferric thiocyanate assay, β‐carotene decolorization assay, determination of the lesion area on mucosa of the stomach	In vitro test—inhibited linoleic acid oxidation Animal model—decreased the ethanol‐induced gastric damage	Kudo et al. (2009)
IF	Alcalase	Antioxidant	Hematoxylin and eosin (HE) staining, Western blotting (Nrf2, some antioxidant proteins)	Animal model—protected kidney from hypertension‐associated renal damage	Tsai et al. (2020)
VSAIW, AIWGA, FVIKP, VMPSTF, FHDPMLR	Alcalase	Antihypertensive	ACE inhibitory activity test	In vitro test—displayed ACE inhibitory activity	Nazir et al. (2020)
Peptide from potato protein autolysate	Autolysis	Antihypertensive	Blood pressure	Animal model—decreased blood pressure	Mäkinen et al. (2016)
ITP, IIP, GQY, STYQT	Thermoase PC10F, Protease S and Proleather FG‐F	Antihypertensive	Blood pressure	Animal model—decreased blood pressure	Ishiguro et al. (2012)
Potato protein hydrolysate, fractions of potato protein hydrolysate	Alcalase	Antidiabetic	Porcine pancreatic α‐amylase inhibition assay	In vitro test—showed porcine pancreatic α‐amylase inhibition	Rahimi et al. (2022)
DIKTNKPVIF	Alcalase	Antidiabetic	Glycosylated hemoglobin (HbA1c), oral glucose tolerance test	Animal model—decreased glycosylated hemoglobin (HbA1c) and glucose level in oral glucose tolerance	Marthandam Asokan et al. (2019)
Potato peptide fractions	Pepsin, Pancreatin, and Chymotrypsin	Anti‐inflammatory	TNF‐α, COX‐2 and iNOS	Cell culture model—Inhibited TNF‐α, COX‐2, and iNOS	Basilicata et al. (2019)
DIKTNKPVIF, IF	Alcalase	Anti‐inflammatory	TLR‐4 and p‐NFκB	Animal model—downregulated TLR‐4 and p‐NFκB	Huang et al. (2020)
DIKTNKPVIF	Alcalase	Anti‐inflammatory	p‐p38 MAPK, FGF‐2, TNF‐α, and IL‐6	Animal model—decreased p‐p38 MAPK, FGF‐2, TNF‐α, and IL‐6	Dumeus et al. (2018)
Potide‐G	Ultrafiltration, DEAE‐cellulose and C_18_‐reversed‐phase HPLC	Antimicrobial	Antibacterial activity, antifungal activity	In vitro test—displayed antibacterial and antifungal activity	Kim et al. (2006)
Potato peptide	Alkaline protease enzyme	Anti‐hyperlipidemic	Serum non‐HDL cholesterol concentration, fecal neutral sterol	Animal model—decreased serum non‐HDL cholesterol and increased fecal neutral sterol excretion	Liyanage et al. (2009)
Proteinase inhibitor‐I Proteinase inhibitor‐II	Ammonium sulfate fractionation, gel filtration, chromatography of gel‐filtered inhibitor on sulfoethyl‐cellulose	Anticancer	UV‐induced AP‐1 activation	Cell‐culture model—inhibited UV‐induced AP‐1 activity	Huang et al. (1997)

##### 3.3.3.1 Antioxidant Activity

Free radicals, including reactive oxygen species and reactive nitrogen species, are produced as a consequence of endogenous physiological metabolism (Devasagayam et al. 2004). These free radicals have the potential to damage cell components (lipid, fat, protein), cell membranes, and DNA, as well as disrupt enzyme activity. When the antioxidant system's capacity to neutralize free radicals is insufficient, conditions such as cancer, pancreatitis, aging, Parkinson's disease, diabetes, and atherosclerosis can occur (Jacob 1995). Both endogenous sources (e.g., glutathione, ubiquinol, uric acid, bilirubin, metalloenzymes such as glutathione peroxidases and superoxide dismutase) and exogenous sources (e.g., vitamin C, vitamin E, and flavonoids) of antioxidants protect the metabolism from free radicals (Jacob 1995). Additionally, food protein hydrolysates and peptides derived from food proteins also exert antioxidant properties (Nwachukwu and Aluko 2019).

Elias et al. (2008) reviewed the potential mechanisms behind the antioxidative properties of protein‐derived peptides (Elias et al. 2008). These mechanisms encompass free radical scavenging, metal ion chelation, interaction with antioxidant enzymes within the body, and reduction of hydroperoxides by specific amino acids such as methionine (Elias et al. 2008). The effectiveness of these antioxidant properties was found to be closely linked to factors such as amino acid composition, molecular weight, and peptide size (Nwachukwu and Aluko 2019).

For example, the antioxidant efficacy (ABTS radical‐scavenging activity) on skeletal muscle cells was notably superior in potato protein hydrolysate compared to potato protein isolate (Chang et al. 2020). Amino acid analysis revealed that His, Phe, and Tyr are the main amino acids in potato protein hydrolysate responsible for ABTS radical cation (ABTS^+•^) scavenging activity (Wu et al. 2020). Furthermore, in terms of Fe (II) chelating capacity, His, Cys, Trp, Asp, and Glu amino acids were suggested to be actively involved (Udenigwe et al. 2016).

In recent years, numerous studies, primarily utilizing in vitro models, have demonstrated the antioxidant activities of potato protein hydrolysates and peptides derived from potato protein (Udenigwe et al. 2016). Various in vitro assays were performed to evaluate the antioxidant capacities of potato protein, potato protein hydrolysate, and potato protein‐derived peptides (Chang et al. 2020; Liu et al. 2003). For instance, patatin, the storage protein in potatoes, displayed 1,1‐diphenyl‐2‐picrylhydrazyl (DPPH) radical scavenging activity and protective properties against peroxynitrite‐mediated dihydrorhodamine 123 oxidation (Liu et al. 2003).

Given that iron is a prominent prooxidative metal, Fe (III) reducing capacity, Fe (II) chelating capacity, and the ferric thiocyanate assays were frequently studied in different studies (Kudo et al. 2009; Udenigwe et al. 2016). Potato protein isolates treated by gastrointestinal proteases exerted Fe (III) reducing capacity and Fe (II) chelating capacity in a dose‐dependent manner (Udenigwe et al. 2016). In another study, the ferric thiocyanate assay was performed to evaluate the antioxidant activity against linoleic acid oxidation of identified bioactive peptides, FGER, FDRR, and FGERR, derived from potato protein hydrolysate. Results demonstrated that the antioxidant activities of FGER, FDRR, and FGERR were 32.1%, 93.0%, and 93.4%, respectively, compared to that of BHA (Kudo et al. 2009).

Udenigwe et al. (2016) showed that potato protein isolates treated by gastrointestinal proteases had the capacity to protect and regenerate glutathione in a dose‐dependent manner in an in vitro model (Udenigwe et al. 2016). This outcome holds significant importance due to the crucial role of glutathione in maintaining metabolic health (Pizzorno 2014), because glutathione has critical roles in protecting metabolism by facilitating the regeneration of vitamins C and E, aiding the transportation of mercury out of cells, neutralizing free radicals, and serving as a cofactor for several antioxidant enzymes (Pizzorno 2014).

Cell culture models were also employed to investigate the antioxidant properties of potato protein hydrolysate and peptides (Chang et al. 2020; Wu et al. 2020). Potato protein hydrolysate obtained through protease treatment (bromelain, Neutrase, and Flavourzyme) of potato protein isolate counteracted the effect of H_2_O_2_ oxidation on the C2C12 murine myoblast cell line (Chang et al. 2020). In another cell culture model, a bioactive peptide derived from potatoes, YFE, was identified and synthesized. This peptide exhibited antioxidant properties against H_2_O_2_ in a Caco‐2 cell culture model. The protection mechanism of this peptide was explained by its ability to increase the activity of endogenous antioxidant enzymes such as catalase, glutathione peroxidase, and superoxide dismutase (Wu et al. 2020).

In addition to in vitro models, the antioxidant capacity of potato‐derived peptides was studied in animal models (Kudo et al. 2009; Tsai et al. 2020). Bioactive peptides derived from potatoes, namely FGER, FDRR, and FGERR, were chemically synthesized and tested for their counteractive effect on ethanol‐induced gastric mucosal damage. Results showed that the administration of these peptides (100 mg/kg body weight of rat) inhibited ethanol‐induced gastric mucosal damage by more than 55% (Kudo et al. 2009). Another animal study showed that IF peptide derived from potato protein hydrolysate protected rats' kidneys from hypertension‐associated renal damage. This protective effect was achieved through the activation of the nuclear factor erythroid 2‐related factor 2 (Nrf2) transcriptional regulator, which plays a crucial role in regulating the expression of antioxidant enzymes (Tsai et al. 2020). Importantly, it was observed that IF peptide induced the expressions of levels of some antioxidant proteins in the kidney, such as glutathione peroxidase 4, heme oxygenase 1, superoxide dismutase 1, superoxide dismutase 2, and peroxiredoxin 2 (PRDX2), to a greater extent compared to captopril, a medication commonly used in the management of hypertension (Tsai et al. 2020).

##### 3.3.3.2 Antihypertensive Activity

Hypertension, affecting one in three people globally, is strongly linked to severe health conditions such as stroke, heart attack, heart failure, and kidney diseases (World Health Organization 2023). Murray et al. (2020) reported that among 87 risk factors, high systolic blood pressure was the leading risk factor for early death worldwide (Murray et al. 2020). Consequently, it is of utmost importance to prioritize prevention and management strategies for hypertension.

In the management of hypertension, angiotensin‐converting enzyme (ACE) inhibitors are frequently prescribed. ACE inhibitors prevent the formation of angiotensin II, which is involved in vasoconstriction, sodium, and water retention (Messerli et al. 2018).

In the Goldblatt rat model of hypertension, oral administration of peptide from autolysis‐digested potato proteins (600 mg/kg body weight) decreased blood pressure in Sprague Dawley male rats (Mäkinen et al. 2016). There are also sweet potato‐based peptides showing antihypertensive effects in in vitro and animal models (Ishiguro et al. 2012; Nazir et al. 2020). Five peptides from sweet potato protein hydrolysates, with the sequences VSAIW, AIWGA, FVIKP, VVMPSTF, and FHDPMLR, were shown to have ACE inhibitory activity in an in vitro test (Nazir et al. 2020). Ishiguro et al. (2012) conducted experiments to assess the effectiveness of commercially available protease enzymes, and three enzymes were chosen (Thermoase PC 10F, Protease S, Proleather FG‐F) for digesting sweet potato proteins. Following the administration of the sweet potato peptides at a dosage of 100/500 mg/kg body weight, spontaneously hypertensive male rats experienced a decrease in blood pressure. This decrease was associated with identified peptides (ITP, IIP, GQY, STYQT) with high ACE inhibitory activity (Ishiguro et al. 2012).

##### 3.3.3.3 Antidiabetic Activity

In 2021, diabetes prevalence was estimated to be 10.5% among individuals aged between 20 and 79 years, with a projected increase to 12.2% by 2045 worldwide (Sun et al. 2022). Diabetes is a serious disease associated with heart attack, chest pain, stroke, cardiovascular diseases, chronic kidney disease, and eye problems (Deshpande et al. 2008). One of the well‐established strategies to manage diabetes is medical nutritional therapy (Sami et al. 2017). Recent studies have shown promising results in the management of diabetes with the use of potato protein hydrolysate and potato protein peptides (Marthandam Asokan et al. 2019; Rahimi et al. 2022).

In an in vitro model, alcalase‐treated potato protein hydrolysate and its fraction were shown to inhibit α‐amylase, which is involved in the hydrolysation of polysaccharides (e.g., starch) into dextrins and maltose (Rahimi et al. 2022). Moreover, an identified peptide (DIKTNKPVIF) from alcalase‐treated potato protein hydrolysate was found to decrease glycosylated hemoglobin (HbA1c) and glucose levels in the oral glucose tolerance test after 4 weeks of treatment in streptozotocin diabetic mice (Marthandam Asokan et al. 2019).

##### 3.3.3.4 Anti‐Inflammatory Activity

Inflammation serves as a vital component of the body's defense system, aimed at safeguarding against harmful agents such as pathogens and toxic chemicals. Inflammation plays a crucial role in promoting the healing process (Furman et al. 2019). However, when the inflammatory response becomes chronic, it transitions from being beneficial to detrimental and is associated with a variety of chronic diseases, including autoimmune diseases, stroke, heart disease, cancer, and non‐alcoholic fatty liver disease. Therefore, controlling chronic inflammation is critical in the management of non‐communicable chronic diseases (Furman et al. 2019). Recent studies have reported that potato protein peptides may play an important role in reducing inflammation levels in the body (Basilicata et al. 2019; Huang et al. 2020; Yan et al. 2014).

Fractionated potato peptides obtained from simulated gastrointestinal digestion of dehydrated potatoes exerted an anti‐inflammatory effect in the intestinal epithelial cell (IEC‐6) model. This effect was observed through the inhibition of tumor necrosis factor‐α (TNF‐α), a powerful pro‐inflammatory cytokine, released from IEC‐6 cells. Moreover, these fractionated potato peptides inhibited the enzyme expressions (cyclooxygenase‐2 (COX‐2) and inducible nitric oxide synthase (iNOS)) in the IEC‐6 model, which are associated with inflammatory diseases of the gastrointestinal tract (Basilicata et al. 2019). In addition to in vitro models, the anti‐inflammatory activity of potato protein peptides was documented in animal models as well (Dumeus et al. 2018; Huang et al. 2020). Intragastric administration of potato peptides, IF and DIKTNKPVIF, at a dosage of 10 mg/kg was tested in two different treatment groups of spontaneously hypertensive rats. In each peptide‐treated group, the protein expression of inflammation markers, such as TLR‐4 and p‐NFκB, was decreased, resulting in decreased protein expression of inflammation markers such as TLR‐4, p‐NFκB, TNF‐α, and IL‐6 in the heart tissue of spontaneously hypertensive rats (Huang et al. 2020). The effect of the DIKTNKPVIF peptide was tested in another animal model. Intraperitoneal administration of synthesized DIKTNKPVIF peptide (15 mg/kg) to SAMP8 mice decreased the high‐fat diet‐induced pro‐inflammatory markers, including p38 MAP kinase, FGF‐2, TNF‐α, and IL‐6, in the liver (Dumeus et al. 2018).

##### 3.3.3.5 Other Potential Health‐Promoting Properties

The benefits of potato protein hydrolysate/peptide extend beyond antidiabetic, antioxidant, antihypertensive, and anti‐inflammatory activities. Additional research has highlighted their potential health‐promoting properties, including antimicrobial (Kim et al. 2006), anti‐hyperlipidemic (Liyanage et al. 2009), and anticancer (Huang et al. 1997) activities. These findings underscore the diverse therapeutic potential of potato protein hydrolysates and peptides in promoting overall health.

#### 3.3.4 Challenges in Commercialization of Potato Bioactive Peptides

Despite the potential health benefits associated with bioactive peptides from potato proteins, several challenges need to be addressed for their commercial application. One of the primary obstacles is the bitter taste of hydrolyzed peptides, which can limit their use in food products. Additionally, the bioavailability of these peptides remains a critical issue, as peptides must survive gastrointestinal digestion and be absorbed into the bloodstream to exert their physiological effects (Martínez‐Medina et al. 2021; Rizzello et al. 2016).

Another challenge lies in the scalability of peptide production. While enzymatic hydrolysis and fermentation methods have proven effective in laboratory settings, the high cost of enzymes and the complexity of large‐scale production present significant barriers. Furthermore, regulatory concerns regarding the safety and efficacy of bioactive peptides in functional foods and nutraceuticals need to be addressed before these peptides can be widely accepted by the food industry (Toldrá et al. 2018; Zhang, Tian, et al. 2021).

Looking ahead, further research is needed to address the main challenges associated with potato‐derived peptides, such as bitterness, stability during gastrointestinal digestion, and scalability of production. Future efforts should focus on optimizing extraction and hydrolysis conditions, as well as improving the bioavailability and sensory properties of the resulting peptides using cost‐effective and sustainable strategies. Additionally, tools such as computational modeling may offer support in understanding structure–function relationships and guiding the design of more effective bioactive peptides from potato byproducts (Wang et al. 2025).

## 4 Other Components of Potato Peel

Potato peels contain approximately 2.5% of total dietary fiber, predominantly in the insoluble fraction (40 g/100 g), which includes cellulose, hemicellulose, and lignin. The fiber content and its composition vary depending on peeling and processing methods; for instance, abrasion peeling yields higher starch and lower lignin content than steam peeling. These fibers, resistant to degradation in the gastrointestinal tract, undergo fermentation in the large intestine, producing short‐chain fatty acids (SCFAs) like acetate, propionate, and butyrate, which contribute to gut health and anti‐inflammatory cytokine production (Blad et al. 2012; Mudgil and Barak 2013). Such characteristics make potato peels an excellent source for fiber extraction and utilization in functional foods.

Dietary fibers derived from potato peels exhibit specific health benefits, including hypocholesterolemic, antidiabetic, and anticancer effects. In animal studies, potato peel fibers lowered plasma cholesterol by 40% and hepatic fat cholesterol by 30% (Al‐Weshahy and Rao 2012; Lazarov and Werman 1996).

In addition to previously mentioned compounds, potato peels are known as a significant source of phenolic compounds, linked to diverse health advantages like antioxidative and antimicrobial effects (Sampaio et al. 2020). These compounds are more concentrated in the peel than in inner tissues, with levels up to ten times higher than in the flesh (Ezekiel et al. 2013; Albishi et al. 2013; Wu et al. 2012). Moreover, certain phenolics involved in the plant's defense mechanism, like caffeic acid, coniferyl alcohol, vanillin, and p‐coumaric acid, are found almost exclusively in the peel (Sampaio et al. 2020).

## 5 Up‐Cycling of Potato Peels in Food Processing

Recent studies have demonstrated the versatility of potato peels as functional ingredients across a wide array of food products, including bakery items, snacks, beverages, and even plant‐based meat substitutes. These applications leverage the high dietary fiber content, antioxidant compounds, and bioactive properties present in potato peels. In bakery products, potato peel powder has been utilized to enhance nutritional profiles, particularly by increasing dietary fiber levels and providing a source of essential minerals and phytonutrients. However, its incorporation also impacts product texture, color, and sensory attributes, necessitating careful optimization of formulation processes (Table 4).

**TABLE 4:** Potato peel applications in food products.

Form of potato peel	Food product	Purpose of application of potato peel	References
Potato peel fiber	Bread	To improve textural properties and extend the shelf life of the product	Curti et al. (2016)
Potato peel powder	Gluten‐free Bread	Increase sensory properties	Jacinto et al. (2020)
	Flatbread	Lowering acrylamide content	Crawford et al. (2019)
	Pasta	Increasing the amount of dietary fiber	Namir et al. (2022)
	Cake	Improve physical (increase volume, weight, and height) and organoleptic properties	Akter et al. 2023
	Cake	Improving textural and sensory properties	Jeddou et al. (2017)
	Chips	Reducing fat content and increasing protein content	Durmaz and Yuksel (2021)
	Cooked rice	Increasing antimicrobial activity against pathogens	Juneja et al. (2018)
	Yogurt	Increasing antioxidant activity, phenolic substance content, consistency, and quality properties	Brahmi et al. (2022)
	Edible film‐coating material	Reducing moisture and oxygen transmission	Rommi et al. (2016)
	Edible filmcoating for cheese	Reducing rancidity and peroxide value	Ma et al. (2022)
	Meatball	Increasing the amount of dietary fiber	Badr and El‐Waseif (2018)
Potato peel extract	Gluten‐free pasta	Increasing antioxidant activity and phenolic substance content	Fradinho et al. (2020)
	Irradiated lamb meat	Reduce oxidation, increase antioxidant activity	Kanatt et al. (2005)
	Sunflower oil	Reduce oxidation, increase antioxidant activity	Saeed et al. (2024)
	Soybean oil	Reduce oxidation, increase antioxidant activity	Franco et al. (2016)
	Soybean oil	Reduce oxidation, increase antioxidant activity	Amado et al. (2014)
	Soybean and sunflower oil	Reduce oxidation, increase antioxidant activity	Mohdaly et al. (2010)
	Edible film‐coating for fish	Maintaining color, juiciness, and hardness	Lopes et al. (2021)
	Edible film‐coating material	Increase antimicrobial and antioxidant activity	Gebrechristos et al. (2020)

In the study where potato fiber was used to improve bread's physicochemical properties by Curti et al. (2016) (Table 4), it was determined that bread samples with 4% potato fiber added were much softer than control samples during storage, and they stated that potato fibers positively affected the textural properties of bread.

In another study, Akter et al. (2023) examined cake samples containing 4%, 6%, and 8% potato peel flour evaluated the effect of potato peel flour on physicochemical, antioxidant, and functional properties as well as the texture and sensory properties. They determined that cake samples containing 8% potato peel flour had the highest values for height, weight, and volume of 2.890 cm, 69.713 g, and 121.240 cm^3^, respectively. In terms of sensory evaluation, cake samples containing 4% potato peel flour received the highest scores. They said that added 4% potato peel flour could produce consumer‐friendly cakes (Table 4).

Namir et al. (2022), as shown in Table 4, enriched the pasta samples with potato peel byproducts. Potato peels contribute not only to the nutritional enhancement but also to the development of innovative textures, such as crispiness in extruded products. Additionally, their antioxidant properties can extend product shelf life by reducing lipid oxidation, a significant concern in high‐fat snack foods. In plant‐based meat substitutes, potato peels serve as binders and structural enhancers, providing texture and moisture retention while maintaining a clean‐label profile that appeals to health‐conscious consumers.

Beyond these uses, potato peels have also been explored in beverage formulations, such as functional drinks, where their phenolic compounds impart antioxidative benefits. The incorporation of potato peel components into food products not only reduces waste generation but also supports innovation by addressing consumer demand for functional, health‐promoting, and sustainable ingredients. This dual approach underscores the potential of potato peels to transform the food value chain, integrating sustainability with product enhancement and fostering a more circular and eco‐friendly food system.

## 6 Evaluation of Potato‐Peel Protein Isolates in Terms of Food Safety

This section evaluates the use of potato peel protein isolates as food additives and their potential in human nutrition with a focus on food safety. Two key groups of bioactive compounds present in potato peels, glycoalkaloids and protease inhibitors, are analyzed due to their toxicological concerns and antinutritional properties.

### 6.1 Glycoalkaloids

Glycoalkaloids are sugar‐derived steroidal alkaloids that serve as secondary metabolites in plants belonging to the *Solanaceae* family, including potatoes, tomatoes, and eggplants (Friedman 2015). These compounds play a crucial role in defending plants against bacteria, fungi, viruses, and other phytopathogens. Found in various plant parts such as blossoms, leaves, roots, and tubers, glycoalkaloids are particularly abundant in potato peels and are produced predominantly during the flowering stage, beginning with seed germination (Friedman 2006).

**FIGURE 2:**
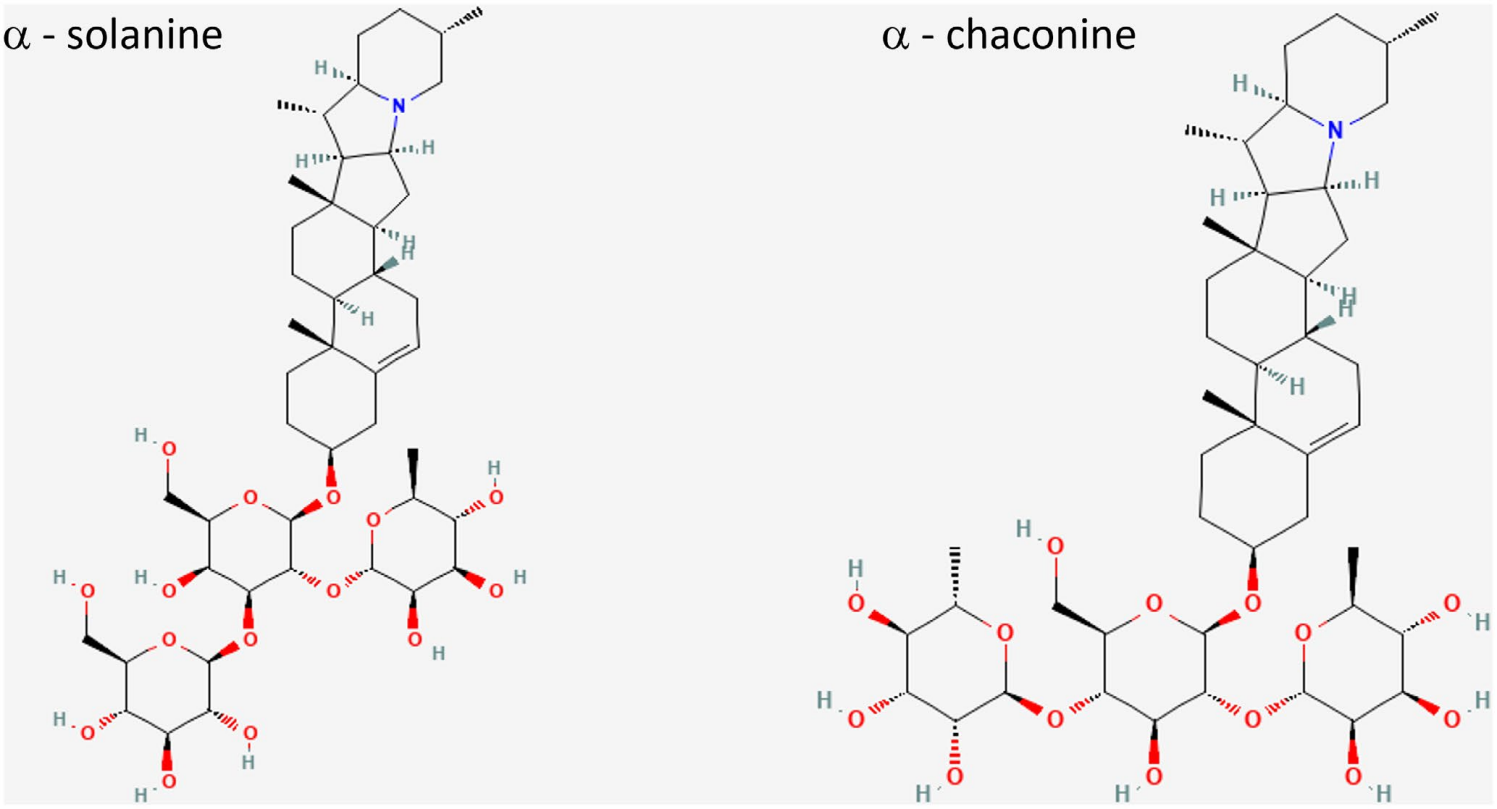
Chemical structure of α‐solanine and α‐chaconine.

The primary glycoalkaloids in potatoes are α‐solanine and α‐chaconine, both derived from the aglycone solanidine (Figure 2). These amphiphilic compounds consist of a hydrophilic carbohydrate chain and a hydrophobic steroidal aglycone core (Winkiel et al. 2022). Their concentrations vary significantly depending on potato cultivar, light exposure, storage conditions, and mechanical damage, with levels ranging from 84 to 3526 ppm (Friedman 2004). Exposure to light during storage, which increases chlorophyll synthesis, can elevate glycoalkaloid levels up to tenfold in potato tubers (Friedman 2006).

#### 6.1.1 Toxicological Concerns

Glycoalkaloids are toxic to humans, animals, and microorganisms. Their toxicity is dose‐dependent, with symptoms including colic, diarrhea, vomiting, and gastroenteritis when consumed in quantities exceeding 3–5 mg/kg body weight (Alves‐Filho et al. 2018; Jimenez‐Champi et al. 2023; Rodríguez‐Martínez et al. 2021). The European Food Safety Authority (EFSA 2020) recommends a daily intake limit of 1 mg/kg body weight to ensure safety. Potato varieties with lower glycoalkaloid content are preferred for consumption, as higher concentrations pose significant risks.

#### 6.1.2 Health Benefits and Therapeutic Potential

Despite their toxicity, glycoalkaloids exhibit various health benefits and therapeutic applications when used within safe limits. Glycoalkaloids, particularly α‐solanine and α‐chaconine, have shown potential in inhibiting cancer cell proliferation and inducing apoptosis in various cancer types, including bladder, gastric, and colorectal cancers (Dong et al. 2022; Gu et al. 2021; Yan et al. 2020). These compounds also provide anti‐inflammatory and antiallergic properties. They have been reported to reduce inflammation and allergic responses, making them valuable for managing hypersensitivity and inflammatory conditions (Friedman 2006). Glycoalkaloids possess antimicrobial properties against bacteria, viruses, fungi, and protozoa, contributing to their potential as natural preservatives or pharmaceuticals (Friedman 2006). Evidence suggests that glycoalkaloids can lower cholesterol levels and regulate blood sugar, supporting cardiovascular and metabolic health (Friedman 2015).

#### 6.1.3 Challenges in Extraction and Processing

The extraction of glycoalkaloids presents technical challenges due to their chemical properties. Efficient extraction methods require acidic media to prevent hydrolysis and optimize recovery (Huie 2002). Table 4 presents the glycoalkaloids' content depending on the potato variety, extraction method, and conditions.

Recent advancements in extraction technologies, such as ultrasound‐assisted extraction, microwave‐assisted extraction, and supercritical fluid extraction, have shown promise in improving efficiency and scalability (Apel et al. 2020; Hossain et al. 2014; Jin et al. 2018). For this reason, during protein extraction from potato processing waste, unintentionally, glycoalkaloids may be extracted simultaneously.

Therefore, in studies conducted to obtain protein and/or dietary fiber from potato peels, monitoring of glycoalkaloid levels in raw biomass and final extract is essential to comply with safety standards, such as those recommended by EFSA (2020). Besides, heat treatments, such as cooking, blanching, or frying, can significantly reduce glycoalkaloid content in the final food product, ensuring safer consumption (Fewell and Roddick 1993). Selecting or breeding potato varieties with lower natural glycoalkaloid levels can minimize risks while preserving beneficial properties (Jimenez‐Champi et al. 2023).

Glycoalkaloids are bioactive compounds with dual roles as toxicants and therapeutic agents. While their toxicity requires careful management in food systems, their health benefits offer significant opportunities. Employing advanced processing methods, rigorous monitoring, and controlled usage can ensure safety while harnessing their therapeutic potential.

### 6.2 Protease Inhibitors

Protease inhibitors found in potato tubers are storage proteins rich in sulfur‐containing amino acids. These proteins regulate protease activity within the tuber and play a role in protecting the potato from pathogens (Heibges et al. 2003). Potato protease inhibitors exhibit a broad inhibition spectrum and, except for the carboxypeptidase inhibitor subgroup, can inhibit trypsin and/or chymotrypsin (Pouvreau et al. 2001).

Protease inhibitors can interfere with protein digestion in the gastrointestinal tract, potentially causing digestive issues. However, they also exhibit anticarcinogenic properties and may stimulate the production of cholecystokinin, a hormone that regulates appetite and digestion (Pouvreau et al. 2005).

In the context of food safety, potato peel protein isolates must be carefully evaluated before their direct or indirect inclusion in food formulations. These isolates contain glycoalkaloids, which are toxic at high concentrations, and protease inhibitors, which may impair digestion. In brief, to ensure safety, the intake of glycoalkaloids should not exceed 3–5 mg/kg body weight to avoid adverse health effects. Similarly, protease inhibitors must be reduced or deactivated through appropriate processing techniques.

## 7 Conclusions

The utilization of potato peels offers a promising pathway toward sustainable food systems and innovative health solutions. Rich in high‐quality proteins, bioactive peptides, dietary fibers, and phenolic compounds, potato peels have demonstrated significant potential for developing functional foods and nutraceuticals with health‐promoting properties. The review highlights considerable advancements in the extraction, purification, and characterization of potato peel‐derived components. However, significant challenges remain, including the scalability of production methods, addressing the bitter taste and bioavailability of peptides, and ensuring safety and regulatory compliance. To address these issues, future research should prioritize cost‐effective and scalable approaches such as optimizing enzymatic hydrolysis conditions, improving peptide purification strategies, and evaluating structure–function relationships. Computational tools may also support these efforts by facilitating in silico predictions of enzyme‐substrate interactions and peptide bioactivity. By focusing on practical and sustainable solutions, the full therapeutic and economic potential of potato peel proteins can be more realistically achieved, contributing to both human health and circular bioeconomy goals.

## Author Contributions

**Aytunga Arik Kibar:** conceptualization (lead), project administration (lead), supervision (lead), writing – review and editing (lead). **Özlem Aslan:** writing – original draft (equal). **Halil Dasgin:** writing – original draft (equal). **Emel Önder Fırat:** writing – original draft (equal). **Halil Rıza Avcı:** writing – original draft (equal). **Serhat Koçer:** writing – original draft (equal). **Fatih Tosun:** writing – original draft (equal).

## Ethics Statement

The authors have nothing to report.

## Consent

The authors have nothing to report.

## Conflicts of Interest

The authors declare no conflicts of interest.

## Data Availability

The data supporting this study's findings are available from the corresponding author upon reasonable request.
